# Proven invasive pulmonary mucormycosis successfully treated with amphotericin B and surgery in patient with acute myeloblastic leukemia: a case report

**DOI:** 10.1186/1752-1947-7-263

**Published:** 2013-12-03

**Authors:** Ana Vidovic, Valentina Arsic-Arsenijevic, Dragica Tomin, Irena Djunic, Radoslav Jakovic, Zlatibor Loncar, Aleksandra Barac

**Affiliations:** 1 Clinic of Hematology, Clinical Center of Serbia, Dr KosteTodorovića 2, Belgrade, Serbia; 2 Institute of Microbiology and Immunology, Faculty of Medicine University of Belgrade, Dr Subotica 1, Belgrade, Serbia; 3 Institute of Pulmonary diseases, Tuberculoses and Thorax Surgery, Clinical Center of Serbia, Dr KosteTodorovića 26, Belgrade, Serbia; 4 Clinic for Emergency Surgery, Emergency Center of Serbia, Pasterova 2, Belgrade, Serbia

**Keywords:** Acute myeloblastic leukemia, Early laboratory diagnosis, Invasive pulmonary mucormycosis

## Abstract

**Introduction:**

Invasive mucormycosis (zygomycosis) is the third most frequent fungal infection in patients with hematologic malignancies. It often results in a fatal outcome mainly due to the difficulty of early diagnosis and its resistance to antimycotics.

**Case presentation:**

A 52-year-old Caucasian man was diagnosed with acute myeloblastic leukemia. Following the induction chemotherapy he developed febrile neutropenia. Meropenem (3×1000mg/day) was introduced empirically. A chest computed tomography showed soft-tissue consolidation change in his right upper lobe. A bronchoscopy was performed and the histology indicated invasive pulmonary aspergillosis based on fungal hypha detection. Also, high risk patients are routinely screened for invasive fungal infections using commercially available serological enzyme-linked immunosorbent assay tests: galactomannan and mannan (Bio-Rad, France), as well as anti-*Aspergillus* immunoglobulin G and/or immunoglobulin M and anti-*Candida* immunoglobulin G and/or immunoglobulin M antibodies (Virion-Serion, Germany). Galactomannan showed low positivity and voriconazole therapy (2×400mg/first day; 2×300mg/following days) was implemented. The patient became afebrile and a partial remission of disease was established. After 2 months, the patient developed a fever and a chest multi-slice computed tomography showed soft-tissue mass compressing his upper right bronchus. Voriconazole (2×400mg/first day; 2×300mg/following days) was reintroduced and bronchoscopy was repeated. Histologic examination of the new specimen was done, as well as a revision of the earlier samples in the reference laboratory and the diagnosis was switched to invasive pulmonary mucormycosis. The treatment was changed to amphotericin B colloidal dispersion (1×400mg/day). The complete remission of acute myeloblastic leukemia was verified after 2 months. During his immunerestitution, a high positivity of the anti-*Aspergillus* immunoglobulin M antibodies was found in a single serum sample and pulmonary radiography was unchanged. A lobectomy of his right upper pulmonary lobe was done and the mycology culture of the lung tissue sample revealed *Rhizopus oryzae*. He remained in complete remission for more than 1 year.

**Conclusions:**

Invasive mucormycosis was successfully treated with amphotericin B, surgery and secondary itraconazole prophylaxis. As a rare disease invasive mucormycosis is not well understood by the medical community and therefore an improvement of education about prevention, diagnosis and treatment of invasive mucormycosis is necessary.

## Introduction

Invasive mucormycosis (IMM;zygomycosis) is the third most frequent invasive fungal infection (IFI) in patients with hematologic malignancies, often resulting in a fatal outcome due to the difficulty of early diagnosis and its resistance to antimycotics [1]. IMM is life-threatening and an emerging infection caused by the ubiquitous filamentous fungi from the Glomeromycota phylum, Zygomycota class and Mucorales order, which include, among others, the Mucoraceae family with genera: *Rhizopus*, *Mucor*, *Rhizomucor*, *Lichtheimia* and *Apophysomyces*.

## Case presentation

**Figure 1:**
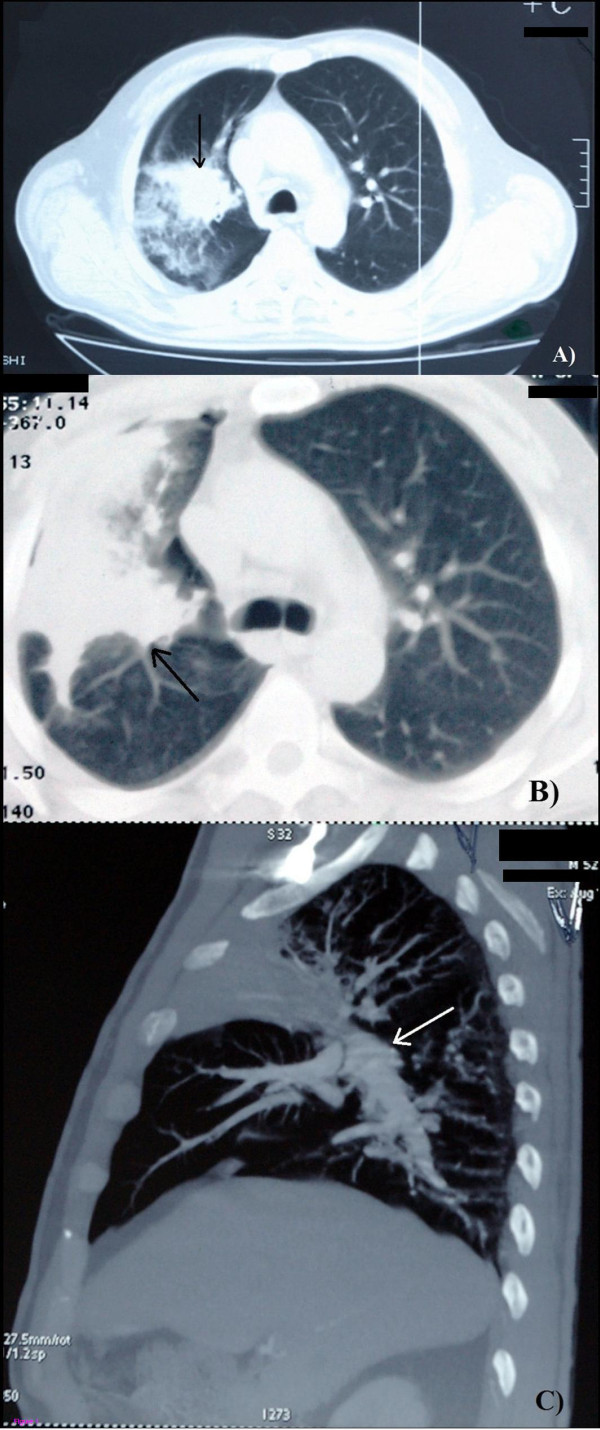
**Findings of pulmonary mucormycosis detected by computed tomography (A, B) and multi-slice computed tomography (C). A)** First hospitalization, soft-tissue alveolar-consolidation changes of the anterior and posterior segment of the upper lobe (arrow). **B)** Second hospitalization, peribronchial circular thickening, with deformation and constriction of all bronchial segments, with peribronchial propagation in the form of irregular, interlobular septal and nodular (up to 1cm) opacities (arrow). Suspected clot masses were viewed in the lumen of the superior vena cava, inferior vena cava, azygos vein and splenic vein. **C)** Third hospitalization, the signs of minor regression of the soft-tissue inflammatory consolidation of the upper right lobe (arrow).

A 52-year-old Caucasian man was diagnosed with acute myeloblastic leukemia (AML) with an intermediary risk group according to the European Leukemia Net [2] in our hospital. Induction chemotherapy was introduced with the “3+7” scheme: Daunoblastina® (daunorubicin) 45mg/kg/body weight (bw) during 3 days and cytosine arabinoside 200mg/kg/bw during 7 days. Post-induction bone marrow aplasia developed on day 4 of the therapy, with a fever (38°C) appearing a day before. Chest X-ray results were normal and meropenem (3×1000mg/day) was empirically introduced. Due to persistent fever, on day 8 of the chemotherapy, caspofungin was empirically introduced intravenously (70mg/first day; 50mg/following days). Also, high risk patients are routinely screened for IFIs using commercially available serological enzyme-linked immunosorbentassay tests: galactomannan (GM) and mannan (Bio-Rad, France), as well as anti-*Aspergillus* immunoglobulin (Ig)G and/or IgM and anti-*Candida* IgG and/or IgM antibodies (Ab; Virion-Serion, Germany). Only one serum sample showed low positivity for GM (index 0.55). On day 14 of therapy, the control chest X-ray showed homogenous opacity of his upper right lobe implying a probable focal pneumonia. Chest computed tomography (CT) verified atelectasis of the lobe with distal pneumonitis (Figure 1A). Bronchoscopy was done on day 31 and histology revealed fungal hyphae suggesting invasive pulmonary aspergillosis (IPA). Voriconazole was introduced (2×400mg/first day; 2×300mg/following days). A control bone marrow biopsy showed partial hematological remission of the AML. On day 44 of hospitalization, the patient was discharged with a normal blood count. On the second hospitalization (1 month later) the patient had no subjective complaints and his blood count was normal. A chest CT showed deformation and constriction of all bronchial segments in his pulmonary upper right lobe. There were interlobular septal and nodular (up to 1cm) opacities. Suspected clot masses were seen in the lumen of the superior vena cava, inferior vena cava, azygos vein and splenic vein (Figure 1B). The patient received re-induction chemotherapy, “3+7” in the same dose, and voriconazole therapy (2×400mg/first day; 2×300mg/following days) was continued as a secondary prophylaxis of IPA. On day 5 of chemotherapy he developed high fever (38.5°C to 40.5°C) and a bronchoscopy was repeated. Direct microscopic examination was done with a bronchoalveolar lavage (BAL) sample, and it was negative.

**Figure 2:**
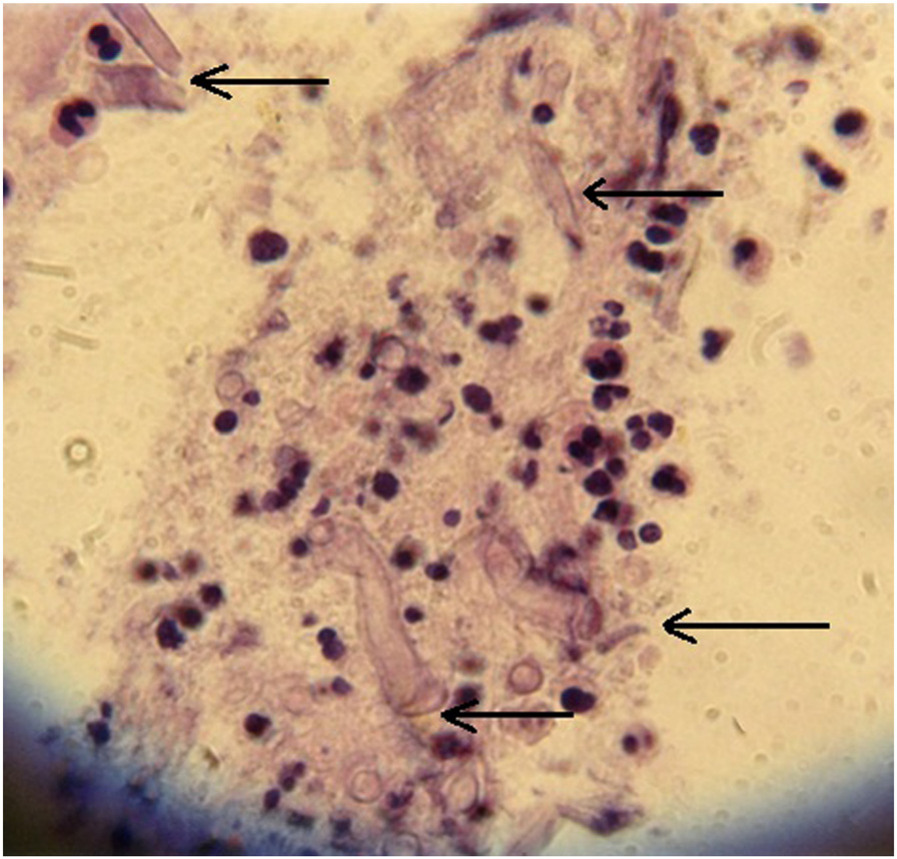
**Cytology findings of pulmonary tissue obtained by biopsy led to diagnosis of mucormycosis; after retesting in a mycology reference laboratory the diagnosis was revised to invasive pulmonary mucormycosis based on wide nonseptic hyphae, suggesting filamentous fungi of the order Mucorales.** Hematoxylin and eosin staining × 400; marked with arrows.

Histologic examination of the new specimen was done, as well as a revision of the earlier samples in the reference laboratory and the diagnosis was switched to pulmonary IMM (Figure 1C). Voriconazole therapy had been stopped and changed to amphotericin B (AmB) colloidal dispersion (ABCD) treatment (1×400mg/day). Concurrently, GM showed low positivity in one serum sample while other biomarkers for IFI were negative. A chest CT, done 2 months after second hospitalization, confirmed a persistence of consolidation of the larger part of his upper right pulmonary lobe. The follow-up bone marrow biopsy showed a complete morphological remission of AML. The patient was discharged and secondary prophylaxis with itraconazole (the only available antimold drug for out-patients in Serbia) (2×200mg/day) was started. On the third hospitalization (after 2 months), the patient complained of cough, blood-streaked sputum, and low fevers. He was in complete remission of AML. A multi-slice chest CT showed signs of minor regression of inflammatory consolidation and the thrombotic masses were not seen (Figure 2). The patient received high-dose consolidation chemotherapy (cytosine arabinoside 3g/m^2^, from D1 to D3). Daily 4 mg/kg/bw of ABCD was reintroduced. The patient developed iatrogenic bone marrow aplasia on day 11 of hospitalization and became febrile. On day 13, hemoptysis, hypotension and septic shock were developed. Manual heart resuscitation and intensive infusion therapy with dopamine hydrochloride infusions were applied (250mg/500mL sodium chloride). A follow-up chest CT was done and showed minor regression of his pulmonary lesions. At the same time IFI early laboratory biomarkers showed a high positive level of anti-*Aspergillus* IgM (420U/mL). A lobectomy of his upper right lobe was done on day 60 during the third hospitalization. Mycology culture of his lung tissue sample revealed *Rhizopus oryzae*. A secondary prophylaxis with itraconazole (2×200mg/day) was done during the next 3 months and the patient remained in remission for more than 1 year.

## Discussion

IMM has emerged as the third most common IFI in patients with hematological malignity and the second most frequent lethal invasive mold infection in those patients [1]. The epidemiological data on IMM are still sparse, thus making determination of the burden of the disease challenging [3]. The incidence of IMM is hard to determine due to a small number of national studies on this problem. The incidence of IMM in United States of America is 1.7 newly diagnosed infections/million [4]. The risk factors of IMM are numerous and very similar to those which predict other mold IFI. The following predisposing factors are of especial significance: long-term neutropenia (>3 weeks), absolute neutrophil count (<200/mm^3^), monocytopenia (<100/mm^3^), graft-versus-host-disease, allogeneic bone marrow transplant, long-term corticosteroid treatment (>1mg/kg/bw), total body iron burden, prolonged hyperglycemia, relapsing and refractory leukemia, previous *Aspergillus* infection, as well as earlier antifungal voriconazole or echinocandin therapy [5]. The key factor for the fast progression of IMM is the angioinvasion by the fungal hyphae, which may cause tissue necrosis and thromboses of the blood vessels, as was shown in our patient [6]. There are six clinical forms of IMM: rhinocerebral, pulmonary, cutaneous, gastrointestinal, disseminated and uncommon presentations [3]. In patients with malignant hemopathies on high-dose combined chemotherapy, the pulmonary form of IMM is seen often and can be followed by dissemination and a lethal outcome [7]. Our patient had key predisposing factors for IMM, such as post-chemotherapy iatrogenic neutropenia and empirical treatment with caspofungin. Even though the diagnostics of IMM is difficult, lacking typical symptoms, signs of disease and specific early laboratory biomarkers, in our case the diagnosis was established by conventional methods and contributed to the proper therapy and a favorable outcome [8].

The definite diagnosis of IMM is hard to establish and often requires invasive procedures such as tissue biopsy and histological examination or *in vitro* culture. Consequently, a diagnosis of IMM is often made postmortem [6]. Timely discrimination between IMM and invasive aspergillosis (IA) is critical for adequate treatment. In relation to CT presentation of IMM and IA, the most suggestive indicator of IMM is the evidence of multiple pulmonary nodules (>10) as well as a pleural effusion. Our patient was initially diagnosed with IPA based on local epidemiology data, low positive GM test, consolidation changes in the lung and the histological proof of fungal hyphae. However, the progression of pulmonary infiltration, development of thrombotic masses in the pulmonary artery, multiple nodule changes in the lung and pleural effusion despite voriconazole treatment led to a revision of the IPA diagnosis, and a new diagnosis, IMM, was proved, which required a well-trained mycologist. The possibility of *Aspergillus* co-infection cannot be excluded, but the chest CT showed that therapy with voriconazole resulted in a progression of infiltration, while the histology and mycology investigations on the lung and BAL samples were negative for *Aspergillus.* In the present context, we speculate that the high levels of anti-*Aspergillus* IgM Ab may be attributed to cross-reactivity with fungal antigens from the Mucoraceae family. It is well known that IgM Ab have potential cross-reactivity among different virus families, several parasites and *Spirochaeta* proteins which could affect the interpretation of some laboratory tests. In spite of that, there is little data about cross-reactivity between different fungal families and further investigation should improve on this.

The treatment of IMM is difficult and fluconazole, voriconazole, flucytosine and echinocandin have negligible effects on IMM. The drug of choice is AmB, as well as lobectomy according to the recommendation of the Expert Medical Board [9], but also posaconazole and itraconazole have potent affectivity on *Rhizopus oryzae*[10]. Combined antifungal therapy is also recommended, usually including AmB and echinocandin or posaconazole [11]. However, surgical treatment is often crucial for IMM [12]. Also, immunotherapy is recommended [13] as well as iron chelating and hyperbaric oxygenation [14,15].

## Conclusions

In our patient, the treatment with ABCD (for 5 weeks), lobectomy, recovery from neutropenia and itraconazole prophylaxis (for 17 weeks) led to recovery from IMM. Also, we take into consideration potential cross-reactivity of Ab between different fungus families. So, a negative serology finding during neutropenia converted to a high positive level of anti-*Aspergillus* IgM Ab during his immune restitution. This fact can confuse medical doctors in making the right decision especially in the absence of specific early laboratory biomarkers for IMM. In conclusion, the “gold standard” for IMM diagnosis is a conventional diagnosis based on lung histology or BAL cytology. IMM, as a rare disease, is not well understood by the medical community. Therefore an improvement of education about prevention, diagnosis and treatment is essential and we believe that the description of this case may contribute to that [8].

## Consent

Written informed consent was obtained from the patient for publication of this case report and accompanying images. A copy of the written consent is available for review by the Editor-in-Chief of this journal.

## Abbreviations

Ab: Antibodies; ABCD: Amphotericin B colloidal dispersion; AmB: Amphotericin B; AML: Acute myeloblastic leukemia; BAL: Bronchoalveolar lavage; Bw: Body weight; CT: Computed tomography; GM: Galactomannan; IA: Invasive aspergillosis; IFI: Invasive fungal infection; Ig: Immunoglobulin; IMM: Invasive mucormycosis; IPA: Invasive pulmonary aspergillosis.

## Competing interests

The authors declare that they have no competing interests.

## Authors’ contributions

AV was responsible for the clinical management of the patient; VAA confirmed diagnoses by histopathology findings and mycology analyses, coordinated and edited the manuscript; DT was responsible for acquisition of data and drafting the manuscript; IDJ performed the radiology analysis; RJ performed the surgery procedure on the patient and drafted the manuscript; ZL was responsible for surgery data; AB performed the laboratory analysis of early biomarkers, designed and edited the manuscript. All authors read and approved the final manuscript.
